# Polymorphic Ventricular Tachycardia Due to Acute Coronary Ischemia: A Case Report

**Published:** 2010-04-01

**Authors:** Praloy Chakraborty, Samanjoy Mukerjee, Rajnish Sardana

**Affiliations:** Department of Cardiology, Metro Hospitals and Heart Institute, New Delhi

**Keywords:** Acute coronary ischemia, Polymorphic VT

## Abstract

Acute myocardial ischemia can cause ventricular tachycardia (VT) in patients with structurally normal heart. Contrary to the fact that in patients with chronic myocardial scarring the ventricular tachycardia is monomorphic, in patients with acute ischemia the ventricular tachycardia is polymorphic and is reversible with coronary revascularization.

We are reporting a 40 year old male who presented with recurrent syncope due to polymorphic ventricular tachycardia in the context of normal QT interval in baseline ECG and normal left ventricular function without any evidence of myocardial injury. Due to recurrent fatal ventricular arrhythmia despite medical management, urgent coronary angiography was done which showed critical obstruction of right coronary artery (RCA). Considering the critical obstruction of RCA responsible for polymorphic VT, emergency PCI of RCA was done. After successful PTCA and stenting to RCA, he had another episode of polymorphic VT which was terminated with intravenous phenytoin. Seven days after the PCI, 24 hours Holter monitoring was done which showed normal sinus rhythm with infrequent ventricular premature complexes and no evidence of VT. He was asymptomatic at six months follow-up.

## Case report

**Figure 1:**
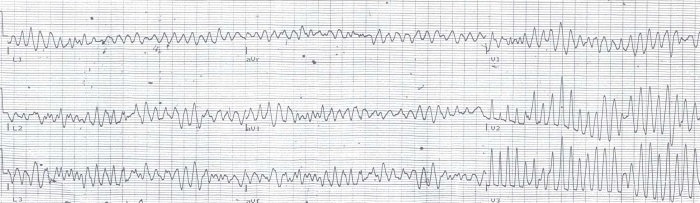
ECG showing polymorphic ventricular tachycardia

**Figure 2:**
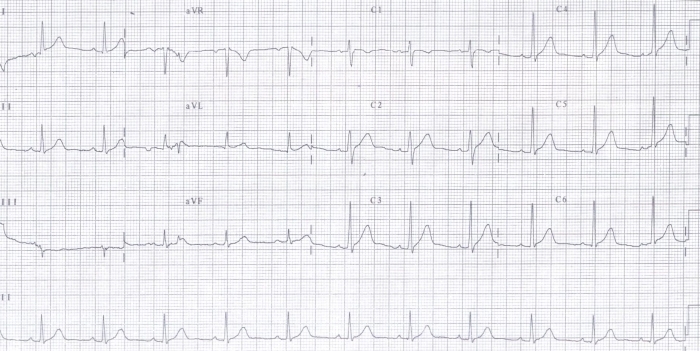
ECG after cardioversion, showing sinus rhythm with QTc of 430 ms

A 40-year-old man presented to the emergency department in a hemodynamically collapsed state due to polymorphic ventricular tachycardia which was treated with direct current shock. Two hours prior to that incident, patient presented to local hospital with history of similar episode of loss of consciousness and 12 lead ECG was done there showing polymorphic ventricular tachycardia (Figure 1) which was treated with intravenous lidocaine.  After direct current shock, ECG in sinus rhythm was normal with QTc of 430 ms (Figure 2). Serum electrolytes were as follows: sodium 140 mmol/L, potassium 3.5 mmol/L, calcium 9.2 mg/dL, magnesium 1.99 mg/dL and cardiac enzymes were negative. Echocardiography showed no regional wall motion abnormality and global left ventricular ejection fraction (LVEF) of 60%.

The patient had history of coronary artery disease. He underwent coronary angiography two years back which showed 60-70% obstruction in the mid part of right coronary artery (RCA) and 50% obstruction in proximal part of left anterior descending coronary artery (LAD). In view of borderline coronary artery disease an exercise test was executed which negated inducible coronary ischemia with good exercise capacity (13.5 METs) and the patient was kept on medical follow up with anti-platelets, statin and beta-blockers. The patient was asymptomatic with medical therapy prior to that event.

With the history of previously documented CAD, normal metabolic parameters, normal QTc and normal LV systolic function, coronary ischemia was thought to be the most probable cause of polymorphic VT and anti-ischemic therapy including beta-blockers and intravenous lidocaine was started.

Despite maximal medical therapy, patient had four episodes of self-terminating polymorphic VT with syncope in next one hour. None of the episodes were preceded by chest pain or any ECG changes suggestive of coronary ischemia.

**Figure 3:**
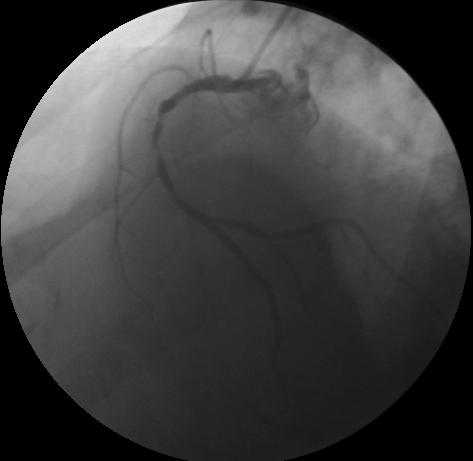
Diffuse obstruction extending from mid to distal right coronary artery (RCA), with maximal obstruction in mid RCA

Due to recurrent potentially fatal ventricular arrhythmia, urgent coronary angiography was done which showed 60% focal obstruction in proximal left anterior descending coronary artery (LAD) and diffuse obstruction extending from mid to distal right coronary artery (RCA), with maximal obstruction in mid RCA (85-90%) (Figure 3).

**Figure 4:**
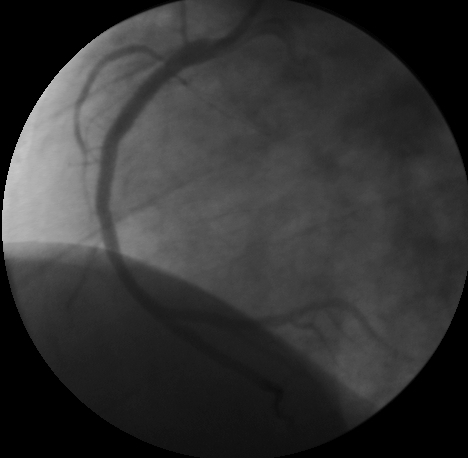
Post PTCA angiogram of RCA

Considering the critical obstruction of RCA responsible for polymorphic VT, emergency PCI of RCA was planned. After balloon dilatation of the obstruction, the RCA was stented with using a 2.75 x 32 mm Xtrm-Track CoCr Stent (Blue medical devices BV) in the mid part and 3 x 23 Xtrm-Track CoCr stent in the proximal part (Figure 4).

One hour after the PCI he again had one episode of self terminating polymorphic VT followed by frequent monomorphic premature ventricular complexes and ventricular bigeminy which was controlled with intra venous phenytoin. There was no episode of polymorphic VT after that.

Patient was kept on dual antiplatelet therapy, statin and betablockers along with IV phenytoin which was stopped after 2 days. Seven days after the PCI, 24 hours Holter monitoring was done which showed normal sinus rhythm with infrequent VPCs and no evidence of NSVT or VT. Echocardiography before discharge showed no RWMA with LVEF of 60%. His follow up period in the coronary care unit was uneventful and he was discharged 10 days after.

## Discussion

Polymorphic VT with a normal QT interval during intervening sinus rhythm is most frequently seen in the context of acute ischemia and may be seen with other cardiac disease states such as cardiomyopathy or HF or in the absence of overt cardiac disease (e.g., idiopathic polymorphic VT, catecholaminergic VT). Although the exact incidence of polymorphic VT in coronary artery disease is not known, coronary artery disease is the most common cause of sudden cardiac death resulting from fatal ventricular arrhythmias [1] and a significant proportion of events occur in subjects without any history of cardiac disease [1,2].

Lethal ventricular tachyarrhythmias in the setting of coronary artery disease results either from acute ischemia or from chronic scar, the mechanisms are different in two different settings. Acute ischemia often occurs in patients without a prior history of heart disease. Although in this setting ventricular fibrillation is the most common terminal rhythm, it is at times preceded by polymorphic ventricular tachycardia [3], as in our case. On the other hand, in patients with impaired left ventricular function, a myocardial scar from a previous infarction may provide the anatomic substrate for reentrant ventricular arrhythmias, manifested most commonly by monomorphic ventricular tachycardia with or without degeneration into ventricular fibrillation.

The incidence of ischemic ST changes before fatal arrhythmia has been observed in 12.6-52% cases [4,5]. Some  investigators have noted increased ventricular ectopic activity during periods of ischemia [6] Active vascular events such as spasm, plaque rupture or thrombosis in the setting of obstructive coronary artery disease precipitate fatal arrhythmias due to acute ischemia [3]. Not only obstructive coronary artery disease but also transient coronary ischemia resulting from coronary vasospasm, anomalous coronary arteries or  myocardial bridges can lead to polymorphic ventricular tachycardia and sudden death [7,8].

Animal studies indicate that within seconds of acute ischemia there is rise in intracellular calcium level and extracellular potassium level [9]. Continued influx of Ca^2+^ may produce after-depolarizations as triggering response for Ca^2+^ dependent arrhythmias. Raised extracellular K^+^ results shortening in of repolarization leading to slow conduction and ultimately to inexcitability. This response is more marked in subepicardium than in subendocardium leading to prominent dispersion of repolarization across myocardium during transmural ischemia. This inhomogeneity and increased dispersion of repolarization results in prolongation of QT dispersion in patients with ischemic heart diseases [10]. Dispersion of conduction and refractoriness favor re-entrant ventricular arrhythmias [11].

Other abnormalities that may contribute to the occurrence of arrhythmias in acute ischemia include alteration of distribution of connexin 43 [12], the production of free fatty acids and oxygen free radicals, acidosis, and an increased catecholamine level [13].

Intravenous lidocaine and beta blockers are useful in treatment of polymorphic VT speci?cally associated with acute myocardial ischemia [14]. Urgent coronary angiography should be considered in the setting of recurrent polymorphic VT when ischemia is suspected [15]. Myocardial re-vascularization may be sufficient therapy in patients surviving polymorphic VT/VF in association with myocardial ischemia when ventricular function is normal and there is no history of an myocardial infarction. However if coronary revascularization is not possible and there is evidence of significant LV dysfunction, the primary therapy for patients resuscitated from polymorphic VT /VF should be an implantable cardioverter defibrillator.
